# Transient Complete Heart Block in a Patient With COVID-19

**DOI:** 10.7759/cureus.15796

**Published:** 2021-06-21

**Authors:** Jia Hong Chen, Bracha Robinson, Palak Patel, Priyaranjan Kata, Anish kumar Kanukuntla, Arthur Okere, Pramil Cheriyath

**Affiliations:** 1 Internal Medicine, Hackensack Meridian Health Ocean Medical Center, Brick, USA; 2 Cardiology, Hackensack Meridian Health Ocean Medical Center, Brick, USA

**Keywords:** covid-19, transient third-degree av block, cardiac manifestations of covid-19, av nodal disturbances, myocardium, transient arrhythmias, viral load

## Abstract

SARS-CoV-2, also known as COVID-19, was first identified in Wuhan, China. Symptoms of COVID-19 are fevers, dry cough and less commonly gastrointestinal (GI) symptoms such as diarrhea that occur in 2 to 14 days of exposure. Infection with COVID-19 leads to hospitalizations due to respiratory compromise. Secondary manifestations of this virus should warrant further investigation since little is known about COVID-19 and its role in the cardiac circuit. We present a patient with COVID-19 who developed transient third-degree AV block initially hospitalized for septic shock. The patient presented with mild symptoms and the transient nature of the complete heart block could be a matter of low viral load in his circulation. He recovered from COVID-19 with no long-term cardiac sequelae. The long-term effects of COVID-19 are still unknown; this case presents the cardiac manifestations of the virus.

## Introduction

In December 2019, a new strain of the coronavirus, SARS-CoV-2, now ubiquitously known as COVID-19, was identified in Wuhan, China. Within a few months, the World Health Organization announced COVID-19 to be a global pandemic, with over 10 million confirmed cases and over 200,000 deaths according to the Center of Disease Control. The most common symptoms of COVID-19 are fevers, dry cough and less commonly GI symptoms such as diarrhea that occur in 2 to 14 days of exposure [1]. COVID-19 is strongly associated with bilateral ground glass opacities seen on a chest x-ray and CT of chest. The severity of this virus has led to soaring hospitalizations as a result of respiratory compromise. In critical conditions, individuals may require mechanical ventilation in the intensive care unit. It is known that the primary infection occurs in the respiratory tract leading to an overwhelming cytokine storm, acute respiratory distress syndrome and multi-system failure.

The raising concern for secondary manifestations of this virus should warrant further investigation. New emerging data with COVID-19 infection has shown cardiac manifestation such as atrial-fibrillation, cardiac arrest and arrhythmias [2,3]. Little is known about COVID-19 and its role in the cardiac circuit. In this case, we present a patient with COVID-19 hospitalized for septic shock in the setting of a complicated diabetic foot infection who developed a transient third-degree AV block.

## Case presentation

**Figure 1:**
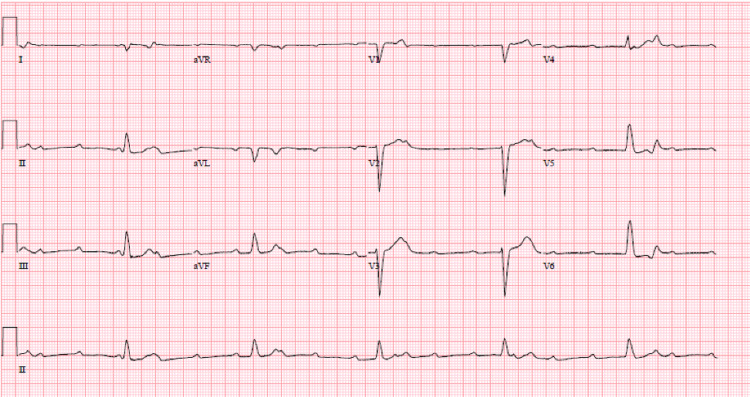
EKG with complete heart block

A 44-year-old male with a ten-day history of a left heel wound presented to the emergency room with a witnessed syncopal episode. He lost consciousness after getting up from a seated position and hit the back of his head. Review of systems was positive for cough and generalized weakness. Vitals signs were temperature of 98.2 °F, heart rate of 34 beats per minute, a blood pressure of 73/42 mm Hg, a respiratory rate of 18 breaths per minute, and a pulse oximetry of 100% on room air. Electrocardiogram showed complete heart block (Figure 1).

Laboratory findings showed a white blood cell count of 17.4, hemoglobin of 9.1, glucose of 193, bicarbonate of 22, C-reactive protein of 7.50, lactic acid of 1.1, and hemoglobin A1c of 8.1. The patient was started on a dopamine drip and transferred to the intensive care unit.

**Figure 2:**
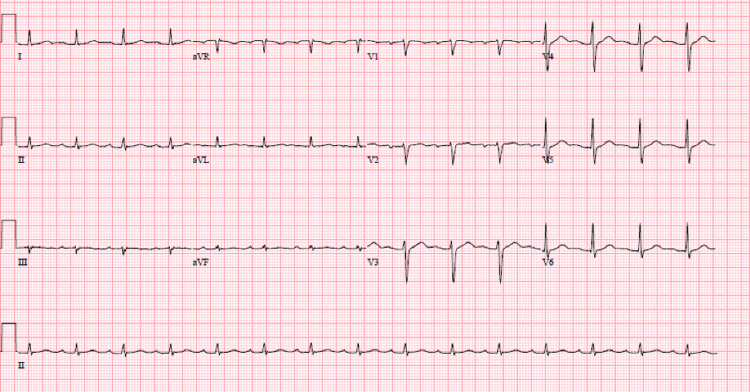
EKG with sinus rhythm and first-degree AV block

Intravenous fluids and broad-spectrum antibiotics were started for septic shock secondary to a diabetic foot infection. The complete heart block was managed by 1 mg of atropine, a maintenance dopamine drip at 20 mcg/kg/min, and pacer pads for transcutaneous pacing. Transvenous pacing was avoided due to his ongoing infection. Overnight, the patient spontaneously converted into sinus rhythm with first degree AV block (Figure 2) at which point the dopamine drip was discontinued. Additional workup included Lyme titers, thyroid stimulating hormone (TSH) level, and COVID-19 screening. The COVID-19 screening was positive, and the other two tests were unremarkable. The patient was not on any AV nodal blocking agents.

The following day an echocardiogram showed an ejection fraction of 70% and mild mitral regurgitation, but was otherwise normal. There were no further episodes of heart block. The patient was discharged several days later in stable condition.

## Discussion

Diseases of the AV node can manifest in patients with underlying structural/ischemic heart diseases, thyroid disease, and insulting infections to the myocardial electrical circuit [4]. It is known that Lyme disease is a common cause of third-degree AV block. The emergence of COVID-19 may add to the etiologies of AV nodal disturbances.

Coronavirus 2 (SARS-CoV-2), the virus that causes COVID-19, affects mammalian cells through invading the angiotensin converting enzyme 2 (ACE 2) receptor expressed in the myocardium [3]. Staggering reports have shown COVID-19 patients may present with arrhythmias, myocarditis and heart failure [2,3].

Recent case reports have shown that patients with COVID-19 developed complete heart block. One case describes a 54-year-old male who developed COVID-19 pneumonia and eventually respiratory failure, requiring intubation. After two weeks of hospitalization, he suddenly had an episode of complete heart block [5]. Another case describes a 77-year-old male with a history of first-degree AV block who presented with symptomatic bradycardia with a heart rate of 30 and complete heart block on his EKG. His rhythm did not resolve, and he required insertion of a permanent pacemaker. He was found to be positive for COVID-19 on routine testing [1]. A 71-year-old female presented with similar symptoms of symptomatic bradycardia and COVID-19 infection. She required permanent pacemaker insertion [6].

There is another case of an 82-year-old male who presented with respiratory failure due to COVID-19. Shortly after intubation he began having complete heart block; he remained in this rhythm for six days. A 55-year-old male with COVID-19 developed multiorgan failure. During the course of his hospitalization, he had several short episodes of second-degree AV block with 2:1 conduction. These episodes resolved spontaneously without intervention. A 43-year-old male with covid-19 was found to have intermittent episodes of complete heart block that started three weeks into his hospitalization. These episodes resolved spontaneously within 24 hours [4]. These case reports show that conduction abnormalities are a rare complication of COVID-19. Sometimes these abnormalities are transient, and other times, require permanent pacemaker insertion.

## Conclusions

COVID-19 can affect the myocardium and cause transient arrhythmias. In this case, we have a 44 y/o male with new onset diabetes and no cardiac history who developed transient complete heart block. The transient nature of the complete heart block could be due to low viral load in his circulation. It is possible that higher viral loads could have more permanent effects. Other mechanisms could be a modest cytokine reaction in the myocardium, not to the extent of myocarditis, which may cause permanent third-degree heart block. The long-term effects of COVID-19 are still unknown; this case presents the cardiac manifestations of this virus in the hope to begin investigating its role in our cardiac conduction system. 
